# Surface Modification on Acoustic Wave Biosensors for Enhanced Specificity

**DOI:** 10.3390/s120912317

**Published:** 2012-09-10

**Authors:** Onursal Onen, Asad A. Ahmad, Rasim Guldiken, Nathan D. Gallant

**Affiliations:** Department of Mechanical Engineering, University of South Florida, 4202 E Fowler Ave, ENB 118, Tampa, FL 33620, USA; E-Mails: onursalonen@mail.usf.edu (O.O.); aaahmad@mail.usf.edu (A.A.A.); guldiken@usf.edu (R.G.)

**Keywords:** bioconjugation, microelectromechanical systems (MEMS), point-of-care, sensor, early detection, ovarian cancer, Bcl-2, surface acoustic wave (SAW), self-assembled monolayer (SAM), polyethylene glycol (PEG)

## Abstract

Changes in mass loading on the surface of acoustic biosensors result in output frequency shifts which provide precise measurements of analytes. Therefore, to detect a particular biomarker, the sensor delay path must be judiciously designed to maximize sensitivity and specificity. B-cell lymphoma 2 protein (Bcl-2) found in urine is under investigation as a biomarker for non-invasive early detection of ovarian cancer. In this study, surface chemistry and biofunctionalization approaches were evaluated for their effectiveness in presenting antibodies for Bcl-2 capture while minimizing non-specific protein adsorption. The optimal combination of sequentially adsorbing protein A/G, anti-Bcl-2 IgG and Pluronic F127 onto a hydrophobic surface provided the greatest signal-to-noise ratio and enabled the reliable detection of Bcl-2 concentrations below that previously identified for early stage ovarian cancer as characterized by a modified ELISA method. Finally, the optimal surface modification was applied to a prototype acoustic device and the frequency shift for a range of Bcl-2 concentration was quantified to demonstrate the effectiveness in surface acoustic wave (SAW)-based detection applications. The surface functionalization approaches demonstrated here to specifically and sensitively detect Bcl-2 in a working ultrasonic MEMS biosensor prototype can easily be modified to detect additional biomarkers and enhance other acoustic biosensors.

## 1. Introduction

Acoustic sensors are capable of measuring physical, chemical and biological quantities using different modes of acoustic (or elastic) waves in various designs and sensor types [1]. They have been investigated and used extensively since the 1970s with the introduction of quartz crystal microbalance (QCM) with a selective adsorptive film on the crystal for chemical sensing [2]. Since then, acoustic sensor technology has been improved and widely used with the advancements in micro-fabrication technologies, enabling high frequency operation (MHz range) with high sensitivity. Acoustic sensors are typically used as delay line devices or resonators, usually along with electrical components. The typical measurement parameters for sensing include, but are not limited to: insertion loss, phase shift, oscillation frequency, quality factor and impedance [1]. Sensing of different measurands is usually accomplished by applied coatings or thin films that are sensitive to target quantity. The selection of these parameters, quantities and the acoustic mode are affected by the sensor type and design. The most typical acoustic sensor types and related acoustic modes are: surface acoustic wave (SAW) sensors (surface acoustic waves), thickness shear-mode (TSM) sensor (resonant thickness shear modes), shear horizontal acoustic plate mode (SH-APM) sensors (bulk shear horizontal waves), and flexural plate-wave (FPW) sensors (lamb waves) [1]. Each sensor type has its advantages and disadvantages depending on the application for optimal operation and sensitivity.

Ovarian cancer is the fifth leading cause of death among women in the United States and the disease has a 1 in 71 lifetime risk [3]. Reduced lethality is associated with diagnosis in earlier stages of the disease progression [3]. B-cell lymphoma 2 protein (Bcl-2) is currently under investigation as a reliable biomarker for ovarian cancer, and it has been shown that urinary Bcl-2 levels are reliably elevated during different stages of ovarian cancer [4,5]. Based on enzyme-linked immunosorbent assay (ELISA) tests using urine samples, the average urinary level of Bcl-2 was found to be 0.59 ng/mL in healthy patients, 1.12 ng/mL in benign disorders, 2.60 ng/mL in early-stage ovarian cancer and 3.58 ng/mL in late-stage ovarian cancer [4].

Based on the reliability of urinary levels of Bcl-2 as a biomarker for detecting ovarian cancer at early stages and distinguishing cancer from other gynecological conditions [4], the development of an ultrasonic biosensor has been undertaken to ultimately be used for point-of-care diagnosis. Toward this objective, the device must be able to quantify the biomarker with high sensitivity with minimal false positive results. The biosensor under development employs shear horizontal surface acoustic waves (SH-SAW) in a delay path configuration for their high sensitivity to surface mass loading and the ability to work under liquid loading [6]. It is comprised of a pair of interdigital transducers (IDTs) microfabricated on ST-cut Quartz wafers in the direction 90° off x-axis. The high sensitivity is achieved due to the high SAW velocity of SH waves and the concentration of the wave energy in the surface. A delay path configuration enables surface modifications to a relatively large surface (compared with micro-size scale sensors) to sense the target quantity. The sensing of Bcl-2 binding in the delay path is achieved by monitoring the oscillation frequency change (or shift) of an oscillatory circuit, in which the sensor is used as the feedback element. In this sensing method, the oscillation frequency is only a function of sensor design and SAW velocity. The mass loading change, in the form of a surface density increase in the delay path, decreases the SAW velocity, leading to a quantifiable decrease in oscillation frequency.

To meet detection and sensitivity performance targets while sensing only mass loading, the delay path must be specifically functionalized to capture only Bcl-2 proteins while also minimizing non-specific adsorption of other proteins or molecules. First, the functional surface of the device (the delay path) must display highly sensitive and specific capture of the Bcl-2 analyte. This is best achieved with the high affinity of an antibody-antigen interaction via an immobilized anti-Bcl-2 capture antibody. The greater the antibody density available on the surface and in the proper orientation for capture, the more sensitive the device will be to small differences in Bcl-2 concentration. Second, a diagnostic device must reliably provide accurate results; false-positives render such devices useless. Therefore, the quantification of only the biomarker must be achieved so efforts to prevent non-specific protein adsorption are critical because any non-Bcl-2 protein bound to the surface will bias the device toward inaccurately higher masses.

The goal of this investigation was to assess the capability of engineered surfaces to enhance antibody density and orientation while reducing non-specific protein adsorption. Strategies including adsorption, covalent bioconjugation, specific protein-protein interactions, and PEG-ylation were employed in various combinations to fabricate candidate surfaces. These candidate surfaces were then compared by ELISA, a common assay for quantifying proteins or making clinical diagnoses. The surface identified by ELISA testing to provide the best combination of sensitivity and signal-to-noise-ratio was fabricated into a SH-SAW device and challenged with a range of Bcl-2 concentrations and another protein to assess sensitivity, selectivity, and performance in a prototype diagnostic sensor. In initial experiments, it was observed that Bcl-2 concentrations were detectable in the range relevant to ovarian cancer [4], even from a protein mixture. Furthermore, only a minimal frequency shift was observed for control solutions with no Bcl-2 present.

These results, especially the challenges by alternative analytes, indicate strong specificity and sensitivity and support the further development of a SH-SAW-based ovarian cancer biosensor utilizing the surface identified here. The surface coating strategies detailed herein are also applicable to the development of other diagnostic devices and biosensors capable of detecting biomarkers for other pathologies. While these surfaces may not work universally, in many instances the development of a new sensor may be as simple as substituting the appropriate capture antibody for a new biomarker. In any case, the findings of this investigation underscore the importance of integrating the design of the active substrate with the mode of detection in the device, which is a principle that should guide the design of any sensor surface.

## 2. Experimental Section

### 2.1. Surface Functionalization

**Figure 1.**
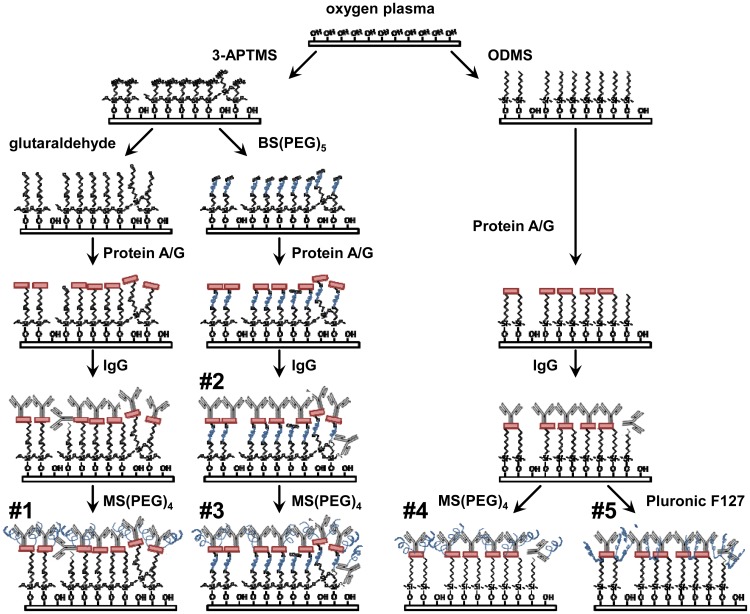
Five surfaces were engineered by sequential assembly with reagents that control the capture antibody orientation and prevent non-specific protein adsorption.

All ELISAs were performed on glass coverslips surface treated according to the following procedures (Figure 1). Similar surface treatments were applied to the delay paths of quartz biosensors in frequency shift tests described below. 9 mm square glass coverslips (Fisher Scientific, Rockford, IL, USA) were exposed to oxygen plasma (Plasma Etch P-50, Carson City, NV, USA) for 5 min at 100 Watts to clean and generate a high density of hydroxyl groups on the surface. Hydroxylated surfaces were functionalized with 3-aminopropyltrimethoxysilane (3-APTMS) (engineered surface assemblies #1-3) or chlorodimethyloctylsilane (ODMS) (#4,5), both purchased from Sigma Aldrich (St. Louis, MO, USA). Anhydrous liquid phase deposition was done in 0.1 M solutions of either alkyl-silane in toluene. The 3-APTMS surface enables further covalent functionalization via amine-reactive compounds, while the ODMS surface strongly promotes protein adsorption through hydrophobic interactions. Alkyl-silane coated coverslips were sequentially rinsed in toluene, ethanol and deionized (DI) water.

3-APTMS surfaces were subsequently exposed to either glutaraldehyde for 2 h (#1) or BS(PEG)_5_ (#2,3) for 15 min. Glutaraldehyde (Fisher Scientific) was prepared 5% w/v in ethanol and 1 M NaOH was used to reach pH 7.4. BS(PEG)_4_ (Thermo Scientific, Rockford, IL, USA) was dissolved in phosphate buffered saline (PBS) for a concentration of 1mM. Both of these reagents act as homobifunctional crosslinkers that react with primary amines. Recombinant Protein A/G (Thermo Scientific) was diluted to 1 mM in PBS and allowed to conjugate (#1-3) or adsorb (#4,5) to each surface for 15 min followed by washing with PBS. Protein A/G has numerous sites with high affinity for the F_c_ portion of antibodies and it is expected to orient the IgG such that the F_ab_ domains will be fully accessible for capture of the analyte [7–9]. All surfaces (#1-5) were exposed to a 5 μg/mL solution of polyclonal rabbit anti-Bcl-2 IgG (Sigma Aldrich) in PBS for 1 h followed by rinsing with PBS. This antibody enables the specific capture of Bcl-2 from test solutions.

To reduce the background, MS(PEG)_5_ (Thermo Scientific) was dissolved in PBS for a concentration of 1mM and samples (#1,3,4) were submerged for 15 min. This amine-reactive molecule is able to bind to available protein or synthetic amines and add PEG chains. Alternatively, samples (#5) were submerged in a 1% w/v solution of Pluronic F127 (Sigma Aldrich) in DI water for 1 h. This poloxamer has a central hydrophobic block that will adsorb to hydrophobic surfaces and flanking PEG blocks that are able to extend from the surface [10]. PEG-ylation provides resistance to protein fouling [11].

### 2.2. Evaluation of Surfaces by Modified Sandwich ELISA

A variation of the sandwich ELISA technique was employed to evaluate the engineered surfaces by using the sensor capture antibody as the bottom of the sandwich configuration. For this application, ELISA is an ideal evaluation method because in addition to capturing the target protein, the surface is challenged with other proteins (the detection antibodies) which will provide an erroneously enhanced signal if non-specifically bound to the surface. This background noise is analogous to the additional mass loading of non-specific protein binding from physiological fluids on an acoustic biosensor.

First the surfaces were challenged with the analyte of interest, Bcl-2, and then detection and enzyme-linked antibodies were applied for quantification. Recombinant human Bcl-2 standard was obtained from R & D Systems (Minneapolis, MN, USA) and diluted to a concentration of 0.1 μg/mL in PBS [4]. Surfaces were incubated with this solution for 1 h and subsequently rinsed with PBS. Monoclonal anti-Bcl-2 antibody was purchased from Santa Cruz (clone 8c8) and used as the detection antibody by incubation on each surface for 1 h at a concentration of 1 μg/mL in PBS. Next, alkaline phosphatase-conjugated anti-mouse IgG obtained from Jackson ImmunoResearch Laboratories (West Grove, PA, USA) was applied to the surface for 1 h at a concentration of 0.3 μg/mL in PBS. Following each of these antibody incubations, the samples were rinsed with 0.05% w/v Tween 20 (Sigma Aldrich) in PBS. Last, the samples were transferred to 24-well plate and 250 μL of a substrate for alkaline phosphatase, *para*-nitrophenyl phosphate (pNPP; Sigma Aldrich), was added to each well. In the presence of alkaline phosphatase, pNPP is converted to a soluble yellow end product. After 30 min, 62.5 μL of 3 M NaOH was added to quench the reaction. The absorbance at 405 nm was measured for solutions sampled from each well using a Synergy HT plate reader (BioTek, Winooski, VT, USA). Control samples with no Bcl-2 protein added were also tested and the signal-to-noise was calculated as the ratio of absorbance with Bcl-2 to absorbance without Bcl-2. It is noted that the units of absorbance are relative and depend on development time and other factors; the trends were used as a measure of performance, but the absolute magnitudes do not correlate to other assays or SAW sensor operation and sensitivity.

### 2.3. SAW-Based Biomarker Detection

A prototype ST-cut Quartz SH-SAW piezoelectric biosensor was fabricated with typical MEMS fabrication techniques in a single mask photolithography process [6]. The sensor was designed to operate at the synchronous frequency of 16.8 MHz. It was employed in an oscillatory circuit, in which the sensor was used as the feedback element. The setup used for characterization involves the biosensor, two variable gain RF amplifiers (Olympus 5073PR and Olympus 5072PR, Olympus NDT Inc., Waltham, MA, USA), a digital frequency counter (Agilent 53220A, Agilent Technologies Inc, Santa Clara, CA, USA), an oscillator (Tektronix TDS2001C, Textronix Inc., Beaverton, OR, USA) and the specifically-designed analog filter. Details of the sensor design, fabrication techniques and experimental setup have been reported elsewhere [6]. The functionalized area of the sensor measured 6.25 mm (delay path length, which is 40 λ) by 12.0 mm (IDT length) [6]. Experiments were performed with a range of Bcl-2 concentrations alone or mixed with bovine serum albumin (BSA; 5 μg/mL in PBS), and compared to PBS or BSA only controls. The change in SAW velocity due to Bcl-2 binding to the delay path was monitored by shifts in the oscillation frequency over 10 min.

### 2.4. Statistics

Pair-wise comparisons between controls and samples exposed to Bcl-2 were analyzed by one-tailed Student's t-tests. P-values less than 0.05 were considered significant. In all ELISA experiments, 3–5 independently fabricated samples of each type were tested and data are presented as mean ± SEM.

## 3. Results and Discussion

Based on preliminary experiments and published bioconjugation methods [12], five engineered surfaces were investigated to (1) maximize the specific analyte capture signal, and (2) minimize the background noise caused by non-specific adsorption of other proteins. The surface assemblies (Figure 1) were composed of reactive or hydrophobic alkyl-silane monolayers, PEG or other bioconjugation agents, Protein A/G for high-affinity orientation of the Bcl-2 antibodies, and covalently linked or adsorbed PEG-ylation reagents to prevent non-specific interactions. The initial plasma oxidation and silane self-assembly fabrication steps for all the assemblies under investigation are compatible with the silica-based SH-SAW devices being developed, as well as sensor applications utilizing silicon, PDMS, or other oxidizable surfaces [13,14].

A modified sandwich ELISA method was used to assess the two major criteria of sensitivity (signal magnitude) and specificity (signal-to-noise) for the five assemblies. The capacity of each substrate to capture the target analyte must be high in order to detect low concentrations and differentiate between cancer and benign conditions in a diagnostic setting. Likewise, the signal-to-noise ratio, represented by the comparison between solutions with and without Bcl-2, is a direct measurement of the specificity of each of the engineered surfaces to capture only the target analyte. The ELISA method is an advantageous evaluation technique because the surface is challenged with other proteins (the detection antibodies) which, if allowed to adsorb, will provide an erroneously enhanced signal similar to the additional mass loading on an acoustic biosensor of non-specific protein binding from physiological fluids.

**Figure 2.**
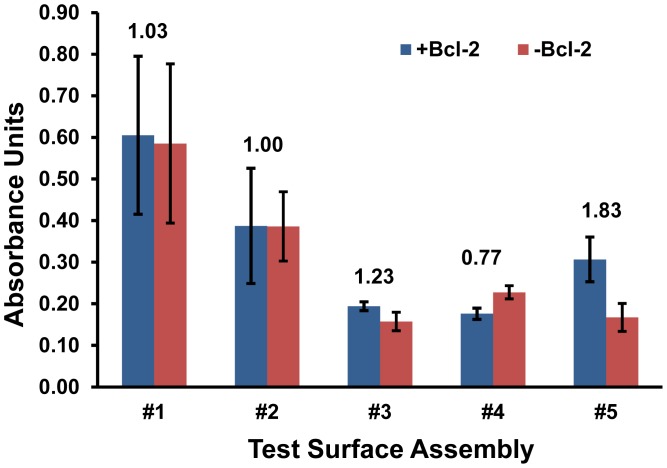
Bcl-2 detection on engineered surfaces was quantified by ELISA. Mean values are presented with error bars representing SEM (n = 3). Signal-to-noise ratios (A_+Bcl-2_/A_-Bcl-2_) are displayed above the bar pairs.

First, the five surface assemblies detailed in Figure 1 were evaluated for their ability to effectively capture Bcl-2 protein from a buffer solution and their ability to resist non-specific mass loading of the subsequently applied antibody solutions that are part of the analysis procedure. The absorbance values produced by all five surfaces exposed to Bcl-2 were quantified, and the comparisons for surface challenges with and without Bcl-2 were used to characterize the signal-to-noise ratios for each assembly (Figure 2).

Surface assemblies #3 and #4 were eliminated based on weak overall signals and inadequate signal-to-noise ratios. Test surface #2 was also eliminated at this stage despite its higher signal, because the background was just as high. Some preliminary conjectures can be made about the possible reasons why these surfaces performed poorly based on their compositions. Assembly #2 lacked any free PEG chains which are known to prevent fouling [15,16], which likely resulted in non-specific protein adsorption. A similar surface with additional PEG-ylation, #3, had a signal-to-noise ratio greater than 1, indicating the effectiveness of MS(PEG)_4_ in reducing background on an amine surface, but its detection capacity was relatively weak. The results for surface #4 indicate the MS(PEG)_4_ was less effective at preventing protein interactions with a hydrophobic surface, and may have even shielded specific Bcl-2 antigen capture since the Protein A/G and anti-Bcl-2 IgG were the only available binders for this amine-reactive compound.

Based on these results, surfaces #1 and #5 were selected for further analysis as the most promising of the five screened initially. Despite its high background (and low signal-to-noise ratio), surface assembly #1 was selected for further investigation over closely related surfaces #2 and #3 because it had the highest overall signal. Similarly #5 was selected over #4 because of its greater signal and the best-in-test signal over background, although its absorbance reading was only half of that produced on surface #1. Interestingly, only #5 relied on the adsorption of a PEG-ylation reagent, Pluronic F127.

Variations of substrates (#1 and #5) were further assessed via ELISA by deconstructing these two surface assemblies. The objective was to either improve the selectivity of #1 or the sensitivity of #5 by eliminating any reagents that were detrimental to performance. The greatest uncertainties were observed in substrate #1 (Figure 2) and this trend continued as variations of #1 were tested. Moreover, the signal-to-noise ratio of #1 with either Protein A/G or MS(PEG)_4_ eliminated was not improved significantly (data not shown). Therefore substrate #1 did not achieve an acceptable threshold for selectivity and was eliminated at this stage.

**Figure 3.**
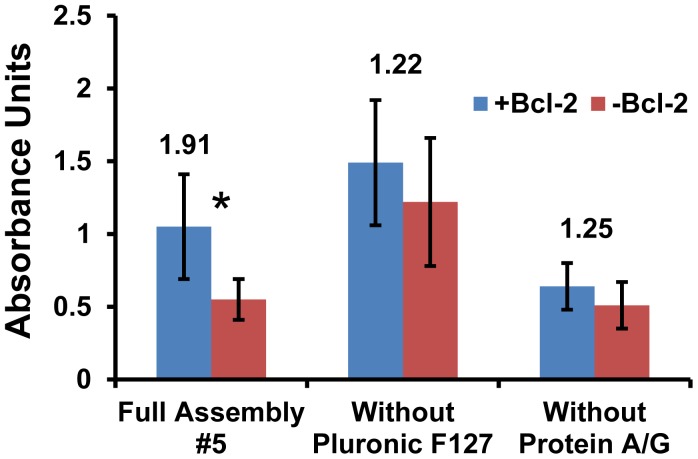
Bcl-2 detection on variations of engineered surface #5 was quantified by ELISA. Mean values are presented with error bars representing SEM (*P < 0.05, n = 4). Signal-to-noise ratios (A_+Bcl-2_/A_-Bcl-2_) are displayed above the bar pairs.

A similar deconstruction of surface #5 for further analysis produced interesting results which clearly demonstrate the important roles of Protein A/G and Pluronic F127 (Figure 3). When the Pluronic F127 was eliminated, the detected signal was increased but the background went up as well. This resulted in a lower signal-to-noise ratio and the difference was no longer statistically significant. For samples where the protein A/G was eliminated, the amount of Bcl-2 detected was lower. This indicates that adsorbing Protein A/G onto a hydrophobic surface more effectively immobilizes and orients the capture IgG to enhance sensitivity when compared to direct antibody adsorption. It should also be noted that F_c_ antibody fragments added before the ELISA did not reduce the non-specific binding of detection antibodies that resulted in higher backgrounds, indicating that the IgGs were not binding appreciably to the Protein A/G via their F_c_ domains and avoiding the need for the expensive F_c_ reagent. Thus, the complete surface assembly #5 displayed the most favorable combination of overall absorbance output and signal-to-noise ratio, which fulfilled both the sensitivity and specificity requirements for the design of an effective diagnostic platform based on surface mass loading.

**Figure 4.**
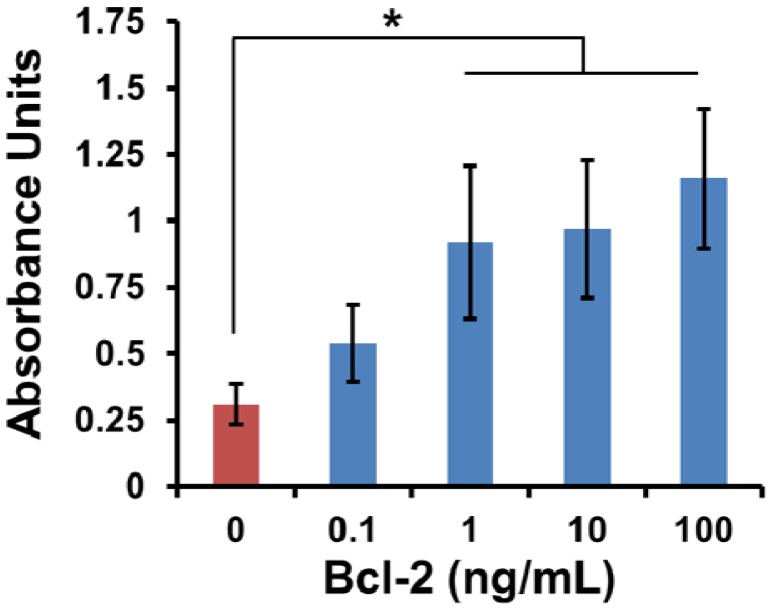
Bcl-2 detection on engineered surface #5 was quantified by ELISA. Mean values are presented with error bars representing SEM (*P < 0.05, n = 5).

Although the ‘best’ surface modification of those tested was identified, a functional biosensor must have the sensitivity to discriminate between low levels of Bcl-2. Up until this point, the Bcl-2 concentration was held constant at 100 ng/mL for each experiment. Since concentrations of the Bcl-2 protein were found to be 2.60 and 3.58 ng/mL respectively in early and late stage ovarian cancer and 0.59 ng/mL in healthy patients [4], detection of early stage ovarian cancer is possible if analyte levels of 1 ng/mL or less can be distinguished. Since it has been demonstrated that mass-loaded sensors possess sensitivity on the order of 0.15 pg/mL [17], it is expected that the surface functionalization will be the limiting factor in sensitive ovarian cancer detection. A range of Bcl-2 concentrations covering four orders of magnitude (0.1–100 ng/mL) were tested and compared to control buffer (Figure 4).

The selected surface #5 displayed highly sensitive Bcl-2 capture over a range of Bcl-2 concentrations. Interestingly, the output from the ELISA did not increase exponentially with the logarithmic series, indicating that consumption of the enzyme substrate was most likely occurring for the higher concentrations on this surface. A significant difference over control was observed for Bcl-2 concentrations of 1 ng/mL or greater. This threshold was important because it is less than the level of Bcl-2 found in the urine of early stage ovarian cancer patients [4]. Bcl-2 at a concentration of 0.1 ng/mL was not distinguishable from control buffer by this ELISA method. Together these results suggest that the surface modification assembly consisting of the hydrophobic ODMS, Protein A/G and Pluronic F127 displays the requisite specificity and sensitivity to capture the Bcl-2 analyte protein, and support its further investigation as a promising substrate for diagnostic sensor applications.

**Figure 5.**
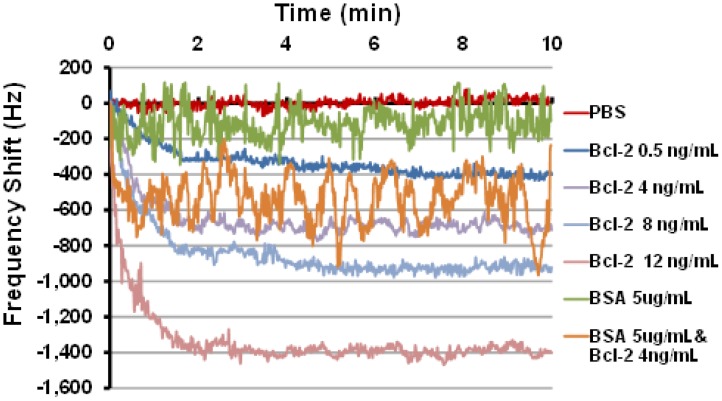
The frequency shifts corresponding to various concentrations of Bcl-2 in PBS, PBS only or BSA controls, and a mixture of Bcl-2 and BSA were measured with a prototype acoustic biosensor.

Last, sensor frequency tests were performed using the oscillatory circuit setup that quantifies the frequency shift caused by changes in mass loading in the delay path. Several tests were performed using this setup with Bcl-2 solutions in PBS with various concentrations. The tests were performed by manually placing 80 μL droplets of Bcl-2 solutions on the delay path ensuring that the shape and coverage of the droplets on the delay path were same in each test. Two controls were also tested: one with PBS only and one with BSA (5 μg/mL) in PBS to ensure specificity of the sensing system for Bcl-2. The frequency shifts as a function of time obtained from these tests are presented in Figure 5, and the mean and uncertainty of the steady state (>2.5 min) frequency shift is summarized in Table 1. It was observed that the frequency response decreased monotonically with increases in Bcl-2 solution concentration. Importantly, when the device designed to detect only Bcl-2 was challenged with a solution of an alternate protein control (BSA), only a minimal frequency change occurred compared to protein free controls. Finally, the sensor was challenged with a mixture of Bcl-2 (4 ng/mL) and BSA (5 μg/mL), chosen to simulate high urine levels of each protein. Even in the presence of 1,000-fold more BSA than Bcl-2, a shift in the frequency similar to Bcl-2 alone was observed, conclusively demonstrating the specificity of this functionalized surface in a biosensor application. Not surprisingly, however, the more highly concentrated protein solutions introduced greater noise, possibly caused by physical interactions between the concentrated but unbound proteins and the multilayered surface which could be reduced by rinsing. Taken together, these results show the best performing surface of those tested has been identified and its specificity and sensitivity demonstrated; however, further optimization and device integration will be necessary before clinical use is feasible.

## 4. Conclusions

This investigation was undertaken to identify a surface modification procedure that would ensure specificity in a biosensor based solely on detecting changes in mass loading from physiological solutions. The functionalization of a SH-SAW device for sensing Bcl-2 was considered here; however these methods may be generalizable to other sensors or biomarkers. A sandwich ELISA method was first employed to quantify the Bcl-2 capture on the substrate and to assess the overall sensitivity and specificity the test assemblies displayed towards the target analyte. Progressively, surfaces displaying substandard capture and signal-to-noise ratio were eliminated until the assembly of ODMS with directly adsorbed Protein A/G and Pluronic was identified as the best of those tested, based on its superior capture efficacy, low background, reproducibility and ease of assembly. The diagnostic relevance of this surface was demonstrated by its ability to detect levels of Bcl-2 comparable to those found in early stage ovarian cancer patients. Finally, this surface modification procedure was tested in a working prototype SH-SAW biosensor, displaying excellent sensitivity and specificity which supports the further development of a point of care ovarian cancer biosensor utilizing the surface identified here.

## Figures and Tables

**Table 1.** Steady state frequency shifts of an acoustic biosensor for Bcl-2.

	**Mean (Hz)**	**Std. Dev. (Hz)**

**PBS**	6	2
**Bcl-2 0.5 ng/mL**	369	37
**Bcl-2 4 ng/mL**	693	29
**Bcl-2 8 ng/mL**	907	40
**Bcl-2 12 ng/mL**	1392	27
**BSA 5 ug/mL**	108	80
**BSA 5 ug/mL & Bcl-2 4 ng/mL**	538	142
